# Emerging COVID-19 variants and their impact on SARS-CoV-2 diagnosis, therapeutics and vaccines

**DOI:** 10.1080/07853890.2022.2031274

**Published:** 2022-02-08

**Authors:** Queenie Fernandes, Varghese Philipose Inchakalody, Maysaloun Merhi, Sarra Mestiri, Nassiba Taib, Dina Moustafa Abo El-Ella, Takwa Bedhiafi, Afsheen Raza, Lobna Al-Zaidan, Mona O. Mohsen, Mariam Ali Yousuf Al-Nesf, Ali Ait Hssain, Hadi Mohamad Yassine, Martin F. Bachmann, Shahab Uddin, Said Dermime

**Affiliations:** a Translational Cancer Research Facility, National Center for Cancer Care and Research, Hamad Medical Corporation, Doha, Qatar; b College of Medicine, Qatar University, Doha, Qatar; c Department of Biomedical Research, Immunology RIA, University of Bern, Bern, Switzerland; d Allergy and Immunology Section, Hamad General Hospital, Hamad Medical Corporation, Doha, Qatar; e Medical Intensive Care Unit, Hamad General Hospital, Hamad Medical Corporation, Doha, Qatar; f Qatar University Biomedical Research Center, Qatar University, Doha, Qatar; g Nuffield Department of Medicine, Jenner Institute, University of Oxford, Oxford, United Kingdom; h Translational Research Institute and Dermatology Institute, Academic Health System, Hamad Medical Corporation, Doha, Qatar

**Keywords:** COVID-19, SARS-CoV-2, Omicron, Coronaviruses, diagnostic testing, vaccine, immunological responses, Viral epidemic

## Abstract

The emergence of novel and evolving variants of SARS-CoV-2 has fostered the need for change in the form of newer and more adaptive diagnostic methods for the detection of SARS-CoV-2 infections. On the other hand, developing rapid and sensitive diagnostic technologies is now more challenging due to emerging variants and varying symptoms exhibited among the infected individuals. In addition to this, vaccines remain the major mainstay of prevention and protection against infection. Novel vaccines and drugs are constantly being developed to unleash an immune response for the robust targeting of SARS-CoV-2 and its associated variants. In this review, we provide an updated perspective on the current challenges posed by the emergence of novel SARS-CoV-2 mutants/variants and the evolution of diagnostic techniques to enable their detection. In addition, we also discuss the development, formulation, working mechanisms, advantages, and drawbacks of some of the most used vaccines/therapeutic drugs and their subsequent immunological impact.

**Key message**
- The emergence of novel variants of the SARS-CoV-2 in the past couple of months, highlights one of the primary challenges in the diagnostics, treatment, as well as vaccine development against the virus.
- Advancements in SARS-CoV-2 detection include nucleic acid based, antigen and immuno- assay-based and antibody-based detection methodologies for efficient, robust, and quick testing; while advancements in COVID-19 preventive and therapeutic strategies include novel antiviral and immunomodulatory drugs and SARS-CoV-2 targeted vaccines.
- The varied COVID-19 vaccine platforms and the immune responses induced by each one of them as well as their ability to battle post-vaccination infections have all been discussed in this review.

## 1. Introduction

An outbreak of pneumonia that began in December 2019 in Wuhan, the capital city of the Hubei Province of China was found to be associated with a novel strain of the Coronavirus that was tentatively named by the WHO as the 2019 novel coronavirus (2019-nCoV). However, on the 11^th^ of February 2020, it was formally renamed as the Severe Acute Respiratory Syndrome Coronavirus 2 (SARS-CoV-2) by the International Committee on Taxonomy of Viruses [1] and the WHO formally named the viral illness as the Coronavirus Disease 2019 (COVID‐19); a disease characterized by respiratory distress, fevers, coughs, fatigue, pneumonia and muscle pain [2–4]. Following the increase in the number of positive infected cases in China, on the 30 January 2020, the WHO declared the viral epidemic a public health emergency of international concern. SARS-CoV-2 is a an enveloped, single-stranded, positive-sense RNA virus belonging to the Betacoronavirus genus in the Coronaviridae family [2,5,6]. This family of viruses was first identified in 1965 by Tyrell and Bynoe and isolated and cultivated from patients with common colds [7]. Viral structural proteins such as the nucleocapsid protein (N), membrane glycoprotein (M), and spike glycoprotein (S) are the primary determinants of virulence and function [8]. Largely like the previous zoonotic coronavirus outbreaks (SARS-CoV and MERS-CoV), the current SARS-CoV-2 virus causes lower respiratory tract infections and may lead to Acute Respiratory Distress Syndromes (ARDS).

**Figure 1.**
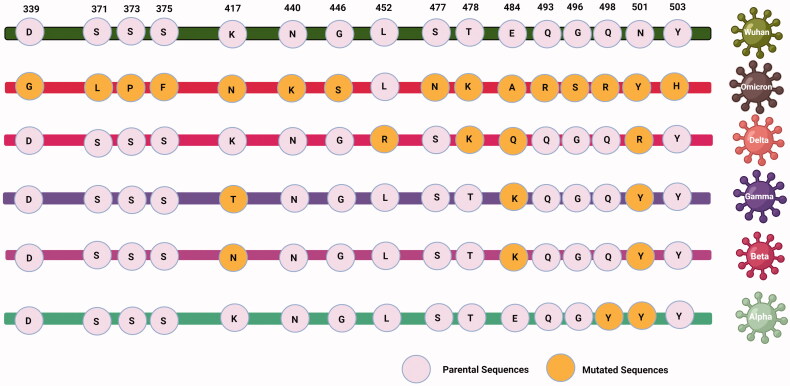
The figure explains about the reported amino acid mutations in RBD region of different SARS- CoV-2 strains.

The emergence of novel variants of the SARS-CoV-2 in the past couple of months highlights one of the primary challenges facing this pandemic. Accumulation of mutations arising out of subsequent viral replication is a natural phenomenon. The SARS-CoV-2 virus is known to evolve at a rate of approximately 1.1 × 10 − 3 substitutions per site per year. This figure corresponds to nearly one substitution every ∼11 days [9]. Although most mutations are found to have no perceivable impact, few mutations were found to give rise to novel high-risk variants of the SARS-CoV-2 virus (Figure 1). The nomenclature and classification of these increasing number of SARS-CoV-2 variants has been a challenge to the WHO. However during late 2020, the WHO prompted the classification of novel SARS- CoV-2 strains as Variants of Interest (VOIs) and Variants of Concern (VOCs) [10]. Specifically, VOIs include variants with mutations that result in changes to receptor binding, reduced efficacy of treatments, decreased neutralization by antibodies and a potential increase in disease severity and/or transmissibility [11]. In addition, VOCs are defined as variants against which there may be strong evidence of an increase in transmissibility, greater disease severity, notable reduction in neutralization by antibodies generated and thus decreased response to treatments and vaccines [11]. (Table 1)

**Table 1.** SARS Cov-2 variants and its impact on transmissibility and treatments.

Variant name	Variant classification	WHO label	Country of Origin /Detection date	Spike protein substitutions	Attributes
B.1.1.7	VOC	Alpha	United Kingdom/ December 2020	69del 70del 144del E484K S494P N501Y A570D D614G P681H T716I S982A D1118H K1191N	↑ Transmissibility (∼50%) ↑ Severity ↑ Case fatality No impact on susceptibility to EUA monoclonal antibody treatments Minimal impact on neutralization by convalescent and post-vaccination sera
B.1.351 B.1.351.2 B.1.351.3	VOC	Beta	South Africa/ December 2020	D80A D215G 241del 242del 243del K417N E484K N501Y D614G A701V	↑ Transmissibility (∼50%) ↓ Susceptibility to EUA monoclonal antibody treatments ↓ Neutralization to convalescent & post-vaccination sera
P.1 P.1.1 P.1.2	VOC	Gamma	Brazil/ January 2021	L18F T20N P26S D138Y R190S K417T E484K N501Y D614G H655Y T1027I	↓ Susceptibility to bamlanivimab/etesevimab monoclonal antibody treatments ↓ Neutralization to convalescent & post-vaccination sera
B.1.617.2 AY.1 AY.2	VOI VOC VOC	Delta	India/ May 2021	T19R V70F T95I G142D E156- F157- R158G (A222V W258L K417N L452R T478K D614G P681R D950N	↑ Transmissibility ↓ Susceptibility to EUA monoclonal antibody treatments ↓ Neutralisation to post-vaccination sera
B.1.427 B.1.429	VOC	Epsilon	California/ July 2020	I4205V D1183Y S13I W152C L452R	↑ Transmissibility (∼20%) ↓ susceptibility to EUA monoclonal antibody treatments ↓ neutralisation to convalescent & post-vaccination sera
B.1.1.529	VOC	Omicron	South Africa\ November, 2021	A67V, del69-70, T95I, del142-144, Y145D, del211, L212I, ins214EPE T547K, D614G, H655Y, N679K, P681H, N764K, D796Y, N856K, Q954H, N969K, L981F	↑ Transmissibility ↑ Risk of re-infection Deletion in the S gene, leading to S gene target failure (SGTF) in some PCR assays. SGTF can be used as a proxy marker to screen for Omicron.
B.1.525	VOI	Eta	United Kingdom/Nigeria December 2020	A67V 69del 70del 144del E484K D614G Q677H F888L	↓ Susceptibility to EUA monoclonal antibody treatments ↓ Neutralization to convalescent & post-vaccination sera
B.1.526	VOI	Iota	United States/ November 2020	L5F D80G T95I Y144- F157S D253G L452R S477N E484K D614G A701V T859N D950H Q957R	↓ Susceptibility to bamlanivimab/ etesevimab monoclonal antibody treatments ↓ Neutralisation to convalescent & post-vaccination sera
B.1.617.1	VOI	Kappa	India/ December 2020	T95I G142D E154K L452R E484Q D614G P681R Q1071H	↓ Susceptibility to EUA monoclonal antibody treatments ↓ Neutralization to post-vaccination sera
C.37	VOI	Lambda	Peru/ August 2020	G75V T76I Δ246-252 L452Q F490S D614G T859N	Unclear data on transmissibility
B.1.621	VOI	Mu	Colombia/ January 2021	R346K E484K N501Y D614G P681H	↑ Transmissibility ↑ Susceptibility to infection
P.3	VOI	Theta	Philippines/ January 2021	E484K N501Y D614G P681H	↑ Transmissibility ↑ Susceptibility to infection

Additionally, in order to synchronize a universal nomenclature that facilitate a streamlined tracking of each of the emerging SARS-CoV-2 variants, the WHO has recommended the use of the Greek Alphabet to uniquely identify each novel variant (Figure 1).

## 2. Advancements in COVID-19 detection & diagnosis

The emergence of novel and evolving variants of SARS-CoV-2 has indeed fostered the need for change in the form of newer and more adaptive diagnostic methods for the detection of SARS-CoV-2 infections. On the other hand, developing rapid and sensitive diagnostic technologies is now more challenging due to emerging variants and varying symptoms exhibited in infected individuals.

SARS-CoV-2 detection technologies mainly target either specific viral nucleic acids (molecular testing), proteins (antigen testing), or anti-SARS-CoV-2 antibodies (serological testing). The choice between each of these tests depends on the selection of right test, right sample and right time [12] as the viral nucleic acid/antigen/antibodies detection varies at different time points during the infection [13].

### 2.1. Nucleic acid-based detection of SARS-CoV-2 infection

Nucleic acid-based detection is now widely used for clinical identification of SARS-CoV-2 infection. Nasopharyngeal swab samples are considered to be the most reliable source for these assays, offering highest sensitivity (97%) as compared to samples obtained from other sources like saliva (85%), nasal swabs (86%) and throat swabs (68%) [14]. Further, the viral RNA load is usually highest between 0 and day 4 of post-symptom infection (89%) and drops to nearly 54% at day 10 to 14. Real-time PCR technology is based on detecting the presence of specific viral RNA belonging to the viral Envelope, Nucleocapsid, Spike and ORF1ab regions. Therefore, viral mutations can potentially alter the accuracy of this method, leading to unpredictable test performances and false-negatives [15]. However, such challenges could be overcome through the use of multi-target assays [14–17]. In addition, studies are now also developing specific primers to enable the rapid detection of VOCs through real-time PCR; For example, a particular group reported the development of PCR primers for the rapid detection of the key mutations in the spike protein of the most recent omicron variant, thus enabling it to be distinguished from other SARS-CoV-2 variants [18]. Another study also described the development of two new PCR- based tests to identify and differentiate the VOCs from regular strains of SARS-CoV-2. These tests are claimed to be comparatively simpler and more rapid than the gold standard methods of genome sequencing [19]. The group also claims that these tests show a strong and reliable correlation to the results obtained through genome sequencing. Apart from these, other groups have also reported the development and use of similar PCR-based tests for detection of novel VOCs [20,21].

In addition, loop-mediated isothermal amplification (LAMP) has also been developed as a rapid, robust and cheap technique that is now considered as a reliable alternative to traditional RT-PCR-based diagnosis [22]. Interestingly, using LAMP, expensive equipment like thermocyclers may be eliminated thereby highlighting the portability of such rapid tests. Moreover, this technique is also highly specific as it uses about 6–8 specific primer sequences to identify eight different regions of the target [13]. Further, Clustered Regularly Interspaced Short Palindromic Repeats (CRISPR) is another novel technology that follows the principle of lateral flow assays. This assay is known to target the E and N genes of SARS-CoV-2. The CRISPR‐Cas13 assays are known to have a sensitivity of greater than 95% and specificity of nearly 99% [23]

In addition, microarray-based technology is also currently being used to detect viral RNA. Here, labelled cDNA molecules synthesized from viral RNA hybridized with solid-phase oligonucleotides on the surface of an array plate are quantified with the help of a microarray plate reader [24].

Next-generation gene sequencing (NGS) methods are also common for the detection of viral presence and helps in understanding the epidemiology of SARS-CoV-2 virus. However, although NGS platforms are accurate and reliable, their practical application is often limited due the involvement of higher costs and expertise [13]. However, whole-genome sequence remains to be the gold standard for the detection of emerging VOCs across the globe. Apparently, since this method is more prolonged and laborious, many studies have come up with faster and similarly robust PCR melting temperature assays that are largely comparable to genome sequencing [25,26]. Interestingly, another group has also reported the development of alternate sequencing platforms based on Sangers sequencing of a single PCR fragment that is capable of identifying and distinguishing all SARS-CoV-2 VOCs that have been identified so far [27].

### 2.2. SARS-CoV-2 antigen and immuno-assay-based detection of SARS-CoV-2 infection

Antigen-based immuno-assays such as immunofluorescent assays, immunochromatographic assays, chemiluminescent immunoassays, and Enzyme Linked Immunosorbent Assays (ELISA) are also reliable methods for the detection of SARS-CoV-2 infections. These commercially available kits are usually compatible with a variety of clinical specimens like nasopharyngeal swabs, nasal swabs, and saliva and mainly detect the presence of two main SARS-CoV-2 antigens (S and N proteins) [28]. However, the success rate of these assays is largely dependent on factors such as disease stage and viral load (1–3 days before to 5–7 days after the onset of symptoms). To address these issues, research on incorporating novel sensor and biosensor technologies, to enhance the sensitivity of these antigen-based immuno- assays is currently ongoing. [13].

### 2.3. Anti-SARS-CoV-2 antibody-based detection of SARS-CoV-2 infection

In contrast with nucleic acids and antigen-based detection techniques, antibody-based techniques are not considered suitable for the early detection of SARS-CoV-2 Infection. This is due to the fact that antibody responses are often generated nearly two weeks post-infection; a time-point at which viral nucleic acid and antigen levels begin to decline [12]. Various binding assays like immunofluorescence, immunochromatographic, chemiluminescence assays and ELISA are used for the detection of antibodies generated specific to the SARS-CoV-2 viral antigen. Most of these kits target the antibodies generated against the viral S and N proteins. Various easy-to-use kits are now available that are based on measuring the ratio between the immunoglobulin M (IgM) and immunoglobulin G (IgG) in the blood. [28]. In addition, humoral immune responses to SARS-CoV-2 can also be detected using simple blotting systems [29]. These are often automated rapid capillary-based platforms through which the reactivity of human IgGs (in serum or plasma samples) against five key SARS-CoV-2 viral antigens [29].

Evidently, the constant development of newer and improved methods for the detection of novel VOCs is of primary importance to keep pace with their rapid emergence. This will also play a key role in monitoring and curbing the spread of the new variants.

## 3. Advancements in COVID-19 preventive & therapeutic strategies

### 3.1. Antiviral and immunomodulatory drugs

Current treatment options for COVID-19 are apparently stratified into two categories; being either antivirals or immune modifiers [30]. In the case of antiviral drugs, Remdesivir has gained sufficient recognition for its ability to contain and manage the viral load and was approved by FDA for the treatment of COVID-19 patients with pneumonia concurrent with the shortage of oxygen supply [30]. It is a broad-spectrum adenosine nucleotide analogue and phosphoramidate prodrug that can target a wide range of viruses includes coronaviruses. The drug mainly functions through the inhibition of replication in the respiratory-associated epithelial cells [31]. According to a recent report, remdesivir triphosphate, being the active form of Remdesivir, resembles the RNA of the coronavirus. Therefore, it is easily integrated into nascent viral RNA strands resulting in halting of viral genome replication [32]. In addition, another study showed that a combination of remdesivir with baricitinib worked better in reducing recovery time of hospitalized patients with COVID-19 pneumonia [33]. Baricitinib is a Janus kinase–STAT signalling inhibitor (JAK-STAT) that possesses antiviral and anti-inflammatory action through the inhibition of clathrin-mediated endocytosis and controls the elevation of cytokine levels. [34].

Moreover, certain anti-HIV drugs like lopinavir and ritonavir that target RNA viruses (retroviruses) were reported to improve the symptoms of patients with SARS [35]. Therefore, they were evaluated for their potential use as a therapeutic agent against COVID-19. However, according to a certain report, no benefit was observed with lopinavir–ritonavir treatment beyond standard care in adult patients hospitalized with severe COVID-19 [36]. Similarly, chloroquine, a drug whose sulphate and phosphate salts have been commercialized as anti-malarial drugs was also shown to be effective against SARS-CoV-2 infections according to a few studies [37–40]. However, a recent trial proved that post-exposure hydroxychloroquine therapy did not prevent SARS-CoV-2 infection in healthy individuals exposed to an infected patient [41]. In addition, other studies have also discredited protease inhibitors like lopinavir and ritonavir and chloroquine to model potent anti-SARS-CoV-2 therapy [42,43].

Recently, two monoclonal antibodies, Tocilizumab and Sarilumab used as anti-inflammatory drugs for rheumatoid arthritis [44] have been repurposed for their use against SARS-CoV-2 Tocilizumab was approved as an immunotherapy drug by FDA for the treatment against the cytokine storm release that is a hallmark of particularly critical COVID-19 infections [30,45]. These monoclonal antibodies function by antagonizing both membrane-bound and soluble interleukin-6 receptors [44], thereby resulting in the blocking of the downstream signal transduction that induces the cytokine release syndrome [46]. Moreover, the clinical trials of the Randomized, Embedded, Multi-factorial, Adaptive Platform Trial for Community-Acquired pneumonia (REMAP-CAP) showed that Tocilizumab and Sarilumab improved survival rate and reduced mortality in hospitalized Covid-19 patients by 28 and 22.2% respectively, when administrated within 24 h of entering intensive care units (ICUs) [30,44,47]. According to the NHS guidance, both drugs are advocated for the treatment of hospitalized Covid-19 patients in ICUs [47].

Further, Casirivimab with Imdevimab forms a unique monoclonal antibody cocktail named REGEN- COV^TM^. These antibodies bind non-competitively to the SARS-CoV-2 spike protein, thus being beneficial in targeting the novel mutant SARS-CoV-2 variants and lowering chances of their immune escape [48]. Results of the phase 3 trial showed that REGEN-COV^TM^ decreased hospitalization or death by 70% in non-hospitalized Covid-19 patients. In addition, it has also been approved by the FDA for the treatment of mild to moderate cases in adults and paediatric Covid-19 patients and in patients at high risk of disease severity [48].

#### 3.1.1. Efficacy of SARS-CoV-2 antiviral drugs on the emerging VOCs

In the wake of the recent emergences of new SARS-CoV-2 variants, it has become increasingly important to evaluate whether the current therapeutics still maintain efficacy against the novel variants. In fact, several *in-vitro* studies were conducted to assess the efficacy of remdesivir against new COVID- 19 variants and more importantly to determine whether these VOCs expressed mutations in the RNA- Dependent RNA Polymerase (RdRP) protein sequence, which is the main target of remdesivir. A recent study conducted on the B.1.1.7 and B.1.351 variants proved that both variants presented a low genetic variation in the RNA replication complex and the most frequent observed substitution was Nsp12 P323L. However, this substitution was not located near the polymerase active site, thus did not affect the inhibition function of remdesivir [49]. In addition, according to Lee et al. the amino acid sequences of the B.1.1.7 and B.1.351 VOCs were found to possess numerous mutations in the spike protein, when compared to the early SARS-CoV-2 strains [50]. However, the amino acid sequence of NSP12, (which possesses RdRp activity), remained to be highly conserved among both the early and novel variants [50]. Moreover, Showers et al. also reported no difference in the antiviral efficacy of remdesivir between early SARS-CoV-2 and these new variants [51]. Furthermore, another study that analyzed the protein sequence of RdRp among SARS-CoV-2 emergent variants show a high conservation in remdesivir- binding residues [52]. Therefore, these reports indicate towards the lack of evidence stating the resistance to remdesivir induced by the VOCs.

Similarly, molnupiravir, a recently FDA approved antiviral drug against SARS-CoV-2 infection is also known to function through targeting the viral polymerase and misdirecting it to incorporate adenosine or guanosine during viral replication, thereby leading to an accumulation of deleterious errors eventually rendering the virus non-infectious [53–55]. Therefore, since reports have proved that the sequences responsible for viral RdRp activity remains to be conserved in early and novel SARS-CoV-2 variants, it is unlikely that the novel VOCs could interfere in the activity of such antiviral drugs. Moreover, other reports also advocate the unrestricted use of the recent FDA approved Paxlovid antiviral drug against the existing VOCs and especially the most recent omicron variant [56]. Therefore, in the light of the above knowledge it may be safe to state that the activity of such antivirals may remain unhindered against the emerging VOCs.

### 3.2. Covid-19 vaccines

**Figure 2.**
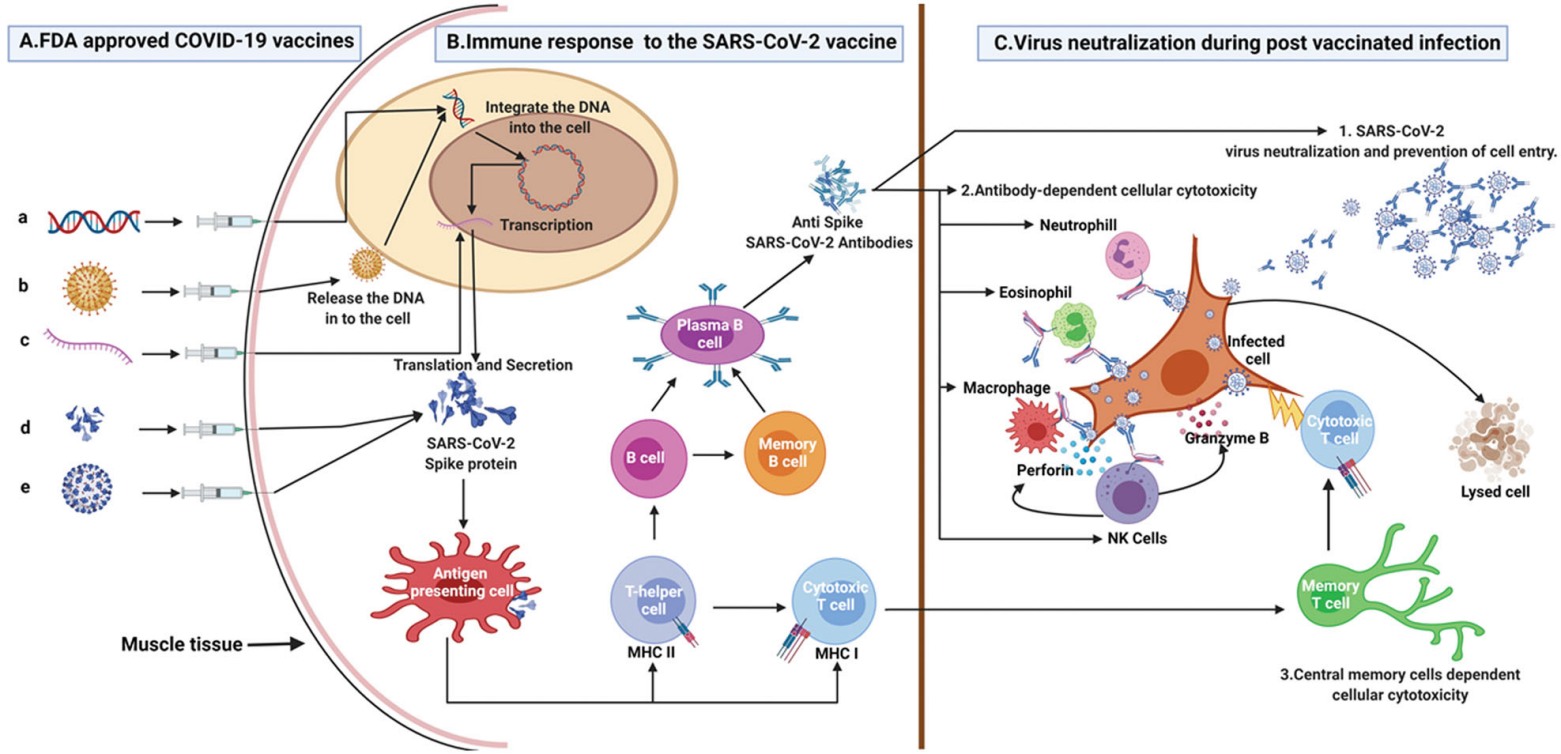
The figure explains about the different COVID-19 vaccine platforms, the immune response to the vaccine and the protective immune response during the post-vaccination infection. (A) Different COVID-19 vaccine platforms. a. DNA vaccine in which SARS-CoV-2 spike open reading frame (ORF) is cloned into a plasmid DNA which will be injected intramuscularly; b. Viral vector platforms in which, the spike protein ORF is cloned into adenovirus genome to form an infectious recombinant virus which will be injected intramuscularly; c. mRNA vaccine, in which SARS-CoV-2 spike mRNA is chemically synthesized and enclosed with lipid nanoparticles then it is injected into human body; d. Protein vaccine in which total or subunit part of spike protein is mixed with specific adjuvant before being injected into human system; e. Inactivated virus vaccine whereby SARS-CoV-2 virus is chemically inactivated, mixed with specific adjuvant then injected intramuscularly. (B) Immune response to the SARS-CoV-2 vaccine: Once in the human body, the different vaccine platforms will synthesize or deliver SARS-CoV-2 total or subunit spike protein which will induce specific memory immune response against SARS-CoV-2 virus. (C) SARS-CoV-2 virus neutralization during post-vaccination infection. 1. If an infection occurs after vaccination, anti-SARS-COV-2 antibodies bind to the SARS-CoV-2 virus and inhibit its attachment to the host cell. 2. Antibody Dependent Cellular Cytotoxicity: The anti-spike antibodies recognize the spike antigen on the infected cells. Four major immune effector cells (neutrophils, eosinophils, macrophages, and NK cells) will recognize the cell bounded antibodies and infected cells and the killing is achieved by cytolytic processes. 3. The memory T cells are quickly converted into cytotoxic T cells and eliminate the infected cells.

In addition to the above elucidated drugs vaccines remain the cornerstone of prevention and protection against infection. Below we discuss the development, formulation, working mechanisms, advantages, and challenges of some of the most used vaccines worldwide (Figure 2). In addition, we also provide an overview of the ongoing trials and cutting-edge research focussed on vaccine efficacy and safety.

#### 3.2.1. Protein subunit vaccines NVX-CoV2373

NVX-CoV2373 (Novavax***)*** is a SARS-COV-2 subunit vaccine constructed from the full-length SARS- COV-2 spike glycoprotein and is produced in the established baculovirus-Spodoptera frugiperda (Sf9) insect cell expression system [57]. This vaccine is formulated through the use of nanoparticles containing trimeric full-length SARS-CoV-2 S glycoprotein adjuvant with saponin based Matrix-M [58]. Studies have shown that this Matrix-M can enhance immune responses by promoting the recruitment, activation, and maturation of central immune cells *via* enhanced antigen presentation and uptake by the antigen presenting cells [59].

The safety and immunogenicity of NVX-CoV2373 was initially tested in a nonhuman primate (baboons and cynomolgus macaque) and mice models. Preliminary results showed that the vaccine elicits a T cell and B cell response, induces a high titre of anti-S IgG and SARS-COV-2 neutralizing antibodies and protects the upper and lower respiratory track from virus infection and pulmonary disease [57,60]. Subsequently, Phase 1-2 clinical trial were conducted to evaluate the safety and immunogenicity of SARS-CoV-2 recombinant S nanoparticle vaccine on humans with or without Matrix-M adjuvant [58]. The outcomes of these trials indicated that the vaccine has a reassuring safety profile and is capable of inducing a robust humoral and T cell immune response [61]. Moreover, the levels of neutralizing antibodies and anti-S IgG detected in vaccinated participants was indeed found to be 4 times higher than those observed in symptomatic COVID-19 outpatient sera [61]. In addition, this vaccine also induces a predominant CD4+ T cell response characterized by high production of IFN-γ, IL-2, and TNF-α. Currently, phase 3 trials are ongoing in 5 different countries (United Kingdom (UK), Northern Ireland, Mexico, Puerto Rico and United States of America) [62]. However, preliminary data from the UK indicates that the efficacy of NVX-CoV2373 is estimated at 89.7% among different subgroups including participants with comorbidities, with no hospitalization or deaths reported in vaccinated individuals [63]. Interestingly, the same study indicates that NVX-CoV2373 has a strong efficacy (86.3%) against the UK variant (B.1.1.7) [63].

#### 3.2.2. Adenovirus vector-based vaccines

##### 3.2.2.1. ZD1222

The AZD1222 (Oxford-AstraZeneca) is a recombinant adenovirus-based SARS-CoV-2 vaccine constructed from the replication-deficient simian chimpanzee adenovirus vector (ChAdOx2) expressing the full- length SARS-COV-2 spike glycoprotein [64]. Chimpanzee vectors are highly suitable for the development of human vaccines due to their high immunogenicity and genome stability that prevents the deletion or mutation of foreign genes [65]. These vectors have been tested in clinical trials of 5000 vaccines (including vaccines for Ebola, malaria, HIV and Crohn disease [66] in which their efficacy to induce a potent CD8+ T cell and antibody responses even with a single dose of the vaccine was reported. [67,68]. In particular, the chimpanzee adenovirus vectors are safe as they avoid issues with pre-existing immunity to human adenoviruses.

The AZD1222 vaccine has been approved by the WHO and is used now in 102 countries [69]. Clinical trials tested on over 60,000 adult participants (aged 18–55 years) in UK, Brazil, South Africa, Kenya, the USA, India and Japan show that the vaccine has a well-tolerated safety profile with no serious adverse events related to the vaccine [70]. After the second dose, most participants were shown to elicit neutralizing antibody responses correlating strongly with anti-spike IgG antibody levels [64,71]. However, the Phase 3 clinical trial interim results from the USA showed that the efficacy of the vaccine could vary according to the immunization regimes (1 or 2 doses) and the length of the interval between the doses (12 or 6 weeks). Such findings support the recent decision in the UK to prioritize use of a 12- week interval between doses [72].

Recently, several European countries suspended the use of the AZD1222 vaccine due to reports linking it to episodes of thrombocytopenia, bleeding, and arterial and venous thromboses occurring within days to weeks after vaccination [73]. According to the European Medicines Agency (EMA), the number of thromboembolic events in vaccinated people is no higher than the numbers seen among the general population [74]. However, rates for venous thromboembolism events observed 28 days after vaccination in Denmark were higher than the expected incidence rates among the general population (50 versus 30) [75]. Altogether, the safety, immunogenicity, and efficacy outcomes of the AZD1222 vaccine are reassuring but these side effects need to be investigated through a large-scale study in different populations to further understand its utility.

##### 3.2.2.2. Gam-COVID-Vac

Gam-COVID-Vac (Sputnik V) is a heterologous adenoviral vector based vaccine against SARS-CoV-2 constructed from two vector components, recombinant adenovirus type 26 (rAd26) and recombinant adenovirus type 5 (rAd5) carrying both the SARS-CoV-2 full-length glycoprotein S gene (rAd26-S and rAd5-S) [76]. Recombinant adenoviruses have been widely used for vaccine development such as hepatitis B, Ebola virus, RSV, HIV and Zika vaccines with an excellent safety profile confirmed in many clinical studies [77–80]. Moreover, recombinant adenovirus vectors elicit robust long-lasting immune response without the need of adjuvant after one or two doses of vaccine [81,82]. The use of 2 different viral vectors will help to overcome any prior anti-adenovirus immunity in the body that may destroy the vector of the second dose [83].

Phase 1/2 clinical trials were conducted to assess safety and immunogenicity of two formulations (frozen and lyophilized) of this vaccine on 76 healthy adult volunteers aged between 18 and 60 years [76]. In Phase 1, participants received a single intramuscular dose of rAd26-S or rAd5-S on day 0 [76]. However, in phase 2, which began no earlier than 5 days after the phase 1 vaccination, participants were administrated a single intramuscular dose of rAD26-S on day 0 followed by another dose of rAD5-S on day 21 [76]. Preliminary data from the phase 1 trials show that no severe adverse reactions were detected in participants after vaccination [76]. Also, the frozen formulation was found to be induce a higher IgG titre (14.703 versus 11.143) and neutralizing antibodies (49.25 versus 45.95) while eliciting higher CD4 (2.5 versus 1.3) and CD8 (1.3 versus 1.1) T cell proliferation rates as compared to the lyophilized formulation indicating the frozen formulation to be more effective than the lyophilized one [76].

Phase 3 clinical trials were performed on a larger scale (nearly 22,000 adults aged at least 18 years) [84]. Participants received 2 doses of the vaccine (dose 1 rAD26-S and dose 2 rAD5-S) or a placebo, 21 days apart [84]. Results of this phase showed that the vaccine efficacy was estimated at 91.6%. In addition, 94% of the participants presented mild adverse reactions, while a minority (less than 0.5%) exhibited severe adverse events [84]. Though four deaths were reported among participants, the cause of death was linked to the vaccine but rather to severe comorbidities [84]. Interestingly, a recent study showed that sera from a donor vaccinated with Gam-COVID-Vac efficiently neutralized the spike protein from B.1.1.7 and B.1.351 strains [85]. This data suggest that this vaccine may offer protection against different SARS-CoV-2 variants.

#### 3.2.3. mRNA vaccines

Since more than a decade, mRNA based therapeutics have raised major interest in cancer and infectious diseases likewise [86]. Particularly, immunization through mRNA vaccines was found to be effective against several viral infections. It has been reported that mRNA vaccines are able to induce potent innate and adaptive immune reactions against Rabies, Zika and Influenza A infections in animal models and in humans [87–90]. Therefore, it is not surprising that mRNA vaccines have now emerged as an effective preventive strategy against SARS-CoV-2 infections. This technology is based on the principle that mRNA is an intermediate messenger that can be easily delivered into host cells and translated into antigen of interest that will trigger a protective antigen-specific immune response in the human body. Within a year from the onset of the COVID-19 pandemic, two mRNA vaccines, namely, BNT162b2 (Pfizer-BioNTech) and mRNA-1273 (Moderna biotechnologies Inc.) were approved by the FDA for emergency use as a prevention against SARS-CoV-2 infection.

Both vaccines, BNT162b2 and mRNA-1273, carry a nucleoside-modified messenger RNA encoding the full-length SARS-CoV-2 spike protein (S) stabilized in the pre-fusion conformation and formulated in lipid nanoparticles (LNPs). These LNPs form a solid lipid complex that encapsulate and stabilize the mRNA and promotes its intracellular uptake [88,91]. While both vaccines are administered intramuscularly in two shots, the second dose is administered after 21 days for BNT162b2 and 28 days for mRNA-1273.

Phase III clinical trials have demonstrated that BNT162b2 and mRNA-1273 vaccines have exhibited more than 90% protection efficacy in people with no prior known infection [92–95]. In fact, Polack et al. demonstrated that the BNT162b2 vaccine conferred 95% protection against COVID-19 in persons 16 years of age and older, with only mild adverse effects that were similar to those observed with other known viral vaccines (short-term fatigue, headache, mild-to-moderate pain at the injection site) [93]. Moreover, the COVE study group in the USA has reported that the mRNA-1273 vaccine has presented 94.1% efficacy in preventing COVID-19 illness with no patterns of safety concerns [92]. In another recent study, Thompson et al. have shown that, for both mRNA vaccines, full immunization with 2 doses of vaccine provides 90% effectiveness against COVID-19 at ≥14 days following the second dose [96]. However, while the mRNA-1273 vaccine is only approved for use in people aged 18 years and older, the BNT162b2 vaccine has been recently granted authorization by the FDA to be used in adolescents aged 12 to 15 years old [97]. Moreover, recent studies have reported that the BNT162b2 vaccine provides strong protection (≥95%) against the COVID-19 variants detected in the United Kingdom (B.1.1.7) and South Africa (B.1.351) [98,99]. Interestingly, a pre-print report has revealed that the effectiveness of BNT162b2 was reduced to 87.9% with the B.1.617.2 COVID-19 variant that has lately emerged in India [100]. As for the mRNA-1273 vaccine, further studies are needed to confirm its effectiveness against the emerging COVID-19 variants.

Evidently, the immunogenic potential of COVID-19 mRNA vaccines have already been documented through various preclinical and clinical trials [89,92,93,101]. Interestingly, a phase I clinical trial on 47 participants demonstrated that the mRNA-1273 induces a robust immune response which was time and dose-dependent [101]. Additionally, while CD4 T cells expression was upregulated in response to the vaccination, only low level of CD8 T cells were detected at the highest tested concentration and after the second vaccination dose [101]. Moreover, another study showed that BNT162b2 induces a broad immune response with SARS-CoV-2 spike-specific neutralizing antibodies and poly-specific CD4+ and CD8+ T cells [102]. Interestingly, the same study has reported a strong memory T cell response up to nine weeks after the booster dose [102].

Apparently, in comparison to other approved vaccines platforms, mRNA-based vaccines have several advantages. For example, in the situation of a widespread global pandemic such as COVID-19, mRNA vaccine production is rapid and can be manufactured on larger scales at relatively lower costs. Moreover, mRNA vaccines are considered safe since they do not contain the full pathogen (unlike vaccines integrating live-vectors or inactivated viruses), and do not carry the viral DNA material that might be associated to genotoxic concerns (like DNA-based vaccines) [88,103].

However, the major problem with mRNA vaccines is the stability of the formulation since they require a strict temperature control for shipment and storage to avoid the degradation of the mRNA. Moreover, the induced activation of the immune system would potentially lead to side effects associated with enhanced inflammatory processes. Therefore, although mRNA vaccines project a powerful strategy to contain the COVID-19 outbreak, more studies are needed to confirm the long-term effectiveness and safety of these vaccines.

#### 3.2.4. Whole virus vaccines

Historically, whole viral inactivation is one of the oldest strategies that have been successfully used to produce vaccines to prevent/treat a variety of viral diseases including influenza, poliomyelitis and human papillomavirus infections [104–106]. In comparison to other whole pathogen-containing vaccines, such as live attenuated virus vaccines, the use of inactivated virus vaccines pose fewer safety concerns, since the pathogen cannot revert to its original state and cause diseases in immunocompromised individuals [107]. Moreover, since they contain the killed pathogen, they can be easily stored and shipped.

According to WHO’s draft landscape of SARS-CoV-2 candidate vaccines, 12 inactivated virus vaccines (14%) are currently in the clinical phase testing. For instance, pharmaceutical companies like Sinovac and Sinopharm, both arising from China have produced inactivated viral vaccines that are currently in phase 3 and 4 of clinical trials respectively [108].

##### 3.2.4.1. COVID-19 vaccine (Vero cell) inactivated

COVID-19 Vaccine (Vero Cell) Inactivated (CoronaVac (formerly PiCoVacc)) developed by Sinovac Biotech Ltd, is an inactivated SARS-CoV-2 (CN2 strain) vaccine adjuvant containing aluminium hydroxide (Al (OH)3) and is administrated through a two-dose regimen (3 µg at day 0 and 28). The virus was extracted from the bronchoalveolar lavage fluid (BALF) of 11 infected patients, cultured in a large- scale Vero cells factories, inactivated with β-propiolactone for 24 h, purified with Ion-Exchange Chromatography (IEC) and Size exclusion Chromatography (SEC) methods and finally adsorbed onto an aluminium hydroxide adjuvant [109]. The vaccine’s safety evaluation has been performed in rhesus macaques (*Macaca mulatta)* monkeys that are known to mimic COVID-19-like symptoms after SARS- CoV-2 infection [110]. Preclinical results using two doses (3 µg and 6 µg) with two immunization schedules (at days 0 and 14 or days 0 and 28) indicated extensive evidences for safety and efficacy, with a complete protection against COVID-19 infection.

In April 2020, the COVID-19 Vaccine (Vero Cell) Inactivated vaccine entered its phase I clinical trial with 144 healthy participants aged between 18 and 59 years old. In most subjects, antibody seroconversion was slightly higher than 75%. These results have been improved to more than 95% in 600 participants enrolled in phase 2 clinical trials [111], with no significant side effects reported . Similar results have been reported in healthy participants older than 60 years old [112]. These results supported the extension of the study to phase III clinical trial using a two-dose regimen (3 µg at day 0 and 28). The clinical trials have been launched in seven countries including Brazil, Indonesia, Turkey, China,

Philippines, Hong Kong, and Chile. Furthermore, two randomized double-blinded placebo control studies have been performed in Brazil and Turkey to determine the efficacy of the vaccine. These trials demonstrated that the COVID-19 Vaccine (Vero Cell) Inactivated efficacy rate for COVID-19 prevention was up to 53% in Brazil and 83% in Turkey [113,114]. In addition, these studies also report that vaccination with COVID-19 Vaccine (Vero Cell) Inactivated induces a humoral response 28 days post-vaccination i.e. 97% neutralizing antibodies against SARS-COV-2 and 99% against RBD-IgG [111,112]. Although only a small number of studies have been published for the COVID-19 Vaccine (Vero Cell) Inactivated vaccine, very few cases of hypersensitivity, including severe allergic reactions (0.009%) have been reported [115,116].

##### 3.2.4.2. Covilo; BIBP-CorV

Covilo; BIBP-CorV (Sinopharm BIBP COVID-19 vaccine) is one of two inactivated virus COVID-19 vaccines developed by Sinopharm. Few studies have investigated the immunogenicity and efficacy of BBIBP-CorV. The first study on BBIBP-CorV has showed that the vaccine induces high levels of neutralizing antibodies in six mammalian species including mice, rats, guinea pigs, rabbits, and non- human primates (cynomolgus monkeys and rhesus macaques). Interestingly, this study reported that two doses of immunization of BBIBP-CorV at 2 mg/dose, is able to provide high protection against SARS- CoV-2 without detectable antibody dependent enhancement of infection [117]. In addition, all macaques in the low and high dose groups did not show a detectable viral load in any lung lobe at 7 days after inoculation of BBIBP-CorV. Also in comparison to the AZD1222 vaccine, both vaccines were found to confer effective protection by preventing the development of viral interstitial pneumonia in all vaccinated macaques [118].

Phase I (192 adults) and phase II (448 adults) clinical trials for the BBIBP-CorV vaccine, have shown that the vaccine is safe and well tolerated at all tested doses. Antibodies were elicited against SARS-

CoV-2 in all vaccine recipients at 42 days after the second dose. These trials included individuals older than 60 that showed significantly high neutralizing antibody titres [119]. Moreover, a particular report also stated that the vaccine had a low rate of adverse reactions and showed high immunogenicity. Yet, however, long-term assessment of safety and efficacy would require phase III trials [120]. The first peer- reviewed data obtained in United Arab Emirates (UAE) and Bahrain showed that BBIBP-CorV is 78.1% effective against symptomatic cases and 100% effective against severe cases [121]. In addition, UAE previously announced interim results showing that the vaccine provided 86% efficacy [122]. Additionally, Sinopharm has started a phase III trial in several countries in Africa [123,124], Asia and Europe [125]. Reports from a randomized, double blind, placebo parallel-controlled phase III clinical trial in Argentina showed that the vaccine portrayed a lower effectiveness (79%) as compared to other approved COVID-19 mRNA vaccines. However, this vaccine has an advantage storage and shipment protocols at regular refrigeration temperatures.

#### 3.2.5. Efficacy of COVID-19 vaccines on the emerging VOCs

Given the alarming frequency of the emergence of SARS-CoV-2 variants, the efficacy of the existing vaccines remains in question. In the light of this, it may be encouraging to state that in addition to the general public, even patients with co-morbidities like cancer and other immuno-compromised individuals like solid organ transplant recipients (SOTRs) have also shown the development of neutralizing antibodies upon vaccination with most conventional vaccines, as discussed in the following reports. According to certain studies, SOTRs may be more commonly associated with breakthrough disease despite being fully vaccinated as compared to the general population [126,127]. Other studies have also reported that most SORTs often develop weak antibody responses against SARS-CoV-2 m-RNA vaccines [128–130]. However, according to another subsequent study even such vulnerable populations are reported to show an increase in neutralizing antibodies against novel VOCs after the third dose of a

SARS-CoV-2 vaccine [131]. In addition, the CAPTURE study also reported the presence of neutralizing antibodies against the novel COVID-19 VOCs in patients with both solid and hematological cancers, upon immunization with the BNT162b2 or AZD1222 vaccine.

Although these studies report that the vaccination-dependent stimulation of an immune response against the novel VOCs in such vulnerable populations is much lowered as compared to normal healthy individuals; yet these results are indeed promising and prove the potency of the currently available vaccines to combat the existing and emerging VOCs.

In addition, the utilization of computational approaches to predict the impact of the VOCs on the vaccines has also proven useful. For example, certain computational approaches like epitope prediction that enables the identification of structural vaccinology targets may also help in modelling the effects of the mutations in the spike proteins observed in the emerging VOCs [132,133]. Moreover, another study has also provided a reliable model for epitope loss in VOCs and their predicted escape from vaccine- induced SARS-CoV-2 antibodies [134]. Such computational approaches prove to be highly useful in foreseeing the impact of the emerging VOCs on the efficacy of COVID-19 vaccines.

## 4. Challenges

Since the SARS-CoV-2 is a novel virus, its invasive properties have not yet been well studied or understood. However, some studies have highlighted a new potential threat in the form of identifying the possible neuro-invasive properties of the novel virus. For example, although the SARS‐CoV and SARS-CoV-2 are both known to enter and infect host cells through ACE2 expressed in the lung cells; according to some studies, ACE2 is not the only receptor that makes host cells susceptible to viral infection. For example, human endothelial and intestinal cells that express ACE2 failed to be infected by SARS-CoV *in vivo* [135,136] On the other hand, cells with comparatively undetectable levels of ACE2 (like hepatocytes) were found to be infected by SARS-CoV [137]. Likewise, SARS‐CoV and MERS‐CoV are known to enter the central nervous system where the ACE2 and DDP430 receptors are very low [138]. Similarly, studies on patients have shown the presence of SARS‐CoV particles in the brain of infected individuals thus supporting the neuro-invasive potential of this virus [139–141]. Since SARS-CoV and the SARS-CoV-2 are like each other, it is possible that the latter may also possess such a neuro-invasive potential. Furthermore, in the case of COVID-19, the latency period may be sufficient for SARS-CoV-2 to invade the CNS and destroy the medullary neurons [138]. In support of this theory, some studies [4,142,143] have reported that a few of the COVID-19 patients did have mild neurologic symptoms like headaches and nausea, while another recent study reported that an estimated 88% of severe COVID-19 cases displayed neurologic manifestations such as acute cerebrovascular disease and impaired consciousness [144].

The rapidly mutating virus has also emerged as a matter of great concern. There is sufficient evidence to state that the SARS-CoV-2 virus is capable of rapidly evolving to invade human immune responses as well as gaining the ability to adapt to other hosts in the near future [145]. Therefore, close monitoring of the novel coronavirus is essential to keep the pandemic in check.

Finally, the greatest challenge of the current COVID-19 pandemic is the transmission of the virus to healthcare workers. Studies report that although stringent isolation and quarantine measures are ensured at medical facilities, yet human-to-human transmission of the SARS-CoV-2 is highly common [146,147]. According to recent reports, nearly 41% of the patients were found to be infected in hospital settings, out of which 29% were medical staff [148]. Such transmission in healthcare settings poses a very serious threat and requires rigorous monitoring.

## 5. Conclusion

The outbreak of the COVID-19 pandemic has unquestionably raised a major public health emergency all over the world. The threatening concern is attributed to the transmissibility of the virus and its capacity to rapidly evolve and mutate leading to the emergence of new uncommon strains.

The leap in advancement of better diagnosis, targeted vaccines and therapeutic remedies is sound evidence that scientific understanding, research, and technology is evolving at the pace of the pandemic. Evidently, continued, and consistent research is required to improve our knowledge of key aspects of the viral pathogenesis that can lead to enhanced preventive and therapeutic strategies.

## Data Availability

Data available within the article or its supplementary materials.
